# When Sex Overcomes Motor Phenotype: New Evidence on Cognitive and Neurobehavioral Symptoms in Parkinson's Disease

**DOI:** 10.1002/brb3.70737

**Published:** 2025-08-04

**Authors:** Massimo Favaro, Chiara Longo, Donatella Ottaviani, Alessandra Dodich, Costanza Papagno

**Affiliations:** ^1^ Center for Mind/Brain Sciences CIMeC University of Trento Rovereto Italy; ^2^ Department of Neurology, Santa Chiara Hospital Azienda Provinciale per i Servizi Sanitari (APSS) Trento Italy; ^3^ Department of Neurology Santa Maria del Carmine Hospital Rovereto Italy

**Keywords:** cognition, mood disorders, Parkinson's disease, sex difference

## Abstract

**Background:**

Sex‐based differences in cognitive and behavioral symptoms have been previously reported in Parkinson's disease (PD), as well as the effects of motor lateralization and phenotypes at onset. However, no studies investigated the interaction between these variables.

**Objective:**

We aimed to evaluate whether sex differences interact with motor phenotype and lateralization at the onset of cognitive and neurobehavioral symptoms.

**Methods:**

Data from 304 PD patients (119 women and 185 men) were retrospectively examined, including comprehensive neurologic, neuropsychological, and neurobehavioral assessments. MANCOVAs on tests divided based on the results of a principal component analysis were performed to compare cognitive and behavioral performance, considering sex, motor phenotype at onset, and onset lateralization as grouping variables. Analyses were also performed on a subsample of patients (*n* = 200) in which subgroups were balanced in terms of motor and demographic features.

**Results:**

Significant sex effects were found, with females showing higher performance compared to males in verbal long‐term memory (*p* = 0.00003), social cognition (*p* = 0.0001), and naming tasks (*p* = 0.03009). Significant interactions between motor phenotype and sex were found: rigid‐akinetic (RA) females showed higher performance than other groups in a verbal memory task (*p* = 0.0183), and tremor‐dominant (TD) females made more errors than the other groups in an inhibitory control task (*p* = 0.03853). Interestingly, RA females performed better on verbal learning than tremor‐dominant (TD) males (*p* = 0.00911), suggesting that sex effects overcome motor phenotype in this cognitive function. No significant interactions were found between sex and lateralization at onset concerning cognitive variables. However, patients with right‐sided onset, in particular females, self‐reported higher levels of behavioral symptoms.

**Conclusions:**

These results emphasize the complex relationship between demographic and PD motor features in delineating the clinical phenotype, which should be considered in designing patient‐tailored strategies for disease monitoring and intervention.

## Graphical abstract

Patients with Parkinson's disease (PD) show variability in clinical manifestations, with symptoms depending on sex, type, and their lateralization at onset (motor phenotype and lateralization). While these factors have been investigated individually, their interaction has not. Therefore, the aim of our study was to verify how sex differences interact with motor features to affect non‐motor symptoms. To do this, we collected data from 304 patients (119 women and 185 men), who underwent assessment of motor symptoms, cognitive abilities, and mood disturbances. Statistical analyses were performed on cognitive and behavioral performance, considering sex, motor phenotype, and lateralization at onset as grouping variables. We found significant sex differences across multiple cognitive domains: women outperformed men in verbal memory and naming and emotion recognition, while men performed better in visuospatial tasks. In addition, women reported higher levels of depression and anxiety than men. We also found a significant interaction between sex and motor phenotype: females with rigidity at onset (RA onset) showed better performance than other groups in a verbal memory task. Interestingly, this group outperformed males with tremor at onset (TD onset) in verbal memory, suggesting that sex may have a stronger influence than motor phenotype in this cognitive area. Sex and motor phenotype also interact in an inhibitory control task, where TD females made more errors. Right‐side onset—in particular in women—was associated with more depression symptoms compared to the other groups. Overall, these findings show that sex interacts in a complex way with onset motor features. This aspect should be considered for patient‐tailored treatment strategies.

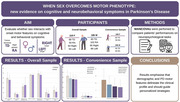

## 1 Introduction

Parkinson's disease (PD) is the second most common neurodegenerative disease, affecting 3% of people over 80 (Lee and Gilbert 2016). PD is characterized by motor symptoms, such as bradykinesia and postural instability (Obeso et al. 2017). These symptoms are typically accompanied at onset by rest tremor (tremor‐dominant [TD] onset phenotype) or rigidity (rigid‐akinetic [RA] onset) (Obeso et al. 2017). Motor symptoms are usually asymmetric, and this asymmetry lasts throughout the course of the disease (Poewe et al. 2017). In addition to motor symptoms, non‐motor disorders significantly contribute to the overall burden of the disease. These symptoms include cognitive impairment, autonomic and sensory dysfunctions, sleep disorders, and neuropsychiatric symptoms, such as depression, anxiety, and apathy (Chaudhuri and Schapira 2009). We will focus on cognitive and behavioral impairments.

Cognitive dysfunction represents one of the most relevant non‐motor features in PD due to its impact on patients and family life (Aarsland et al. 2021).

PD's neurodegenerative processes affect male and female brains differently, leading to distinct clinical expressions (Picillo and Fasano 2015). In fact, the diagnosis of PD in males is 1.5 times more frequent than in females. In addition, the development of motor symptoms in females begins on average 2.2 years later compared to males (Twelves et al. 2003), with tremor being more common both as an initial symptom and throughout the disease and associated with milder motor decline (Haaxma et al. 2007).

Several studies have shown sex differences in cognitive abilities among PD patients. The exact causes remain unknown, even though the underlying mechanisms may involve multiple factors (Heller et al. 2013). Overall, previous literature suggests a domain‐specific sex difference pattern in cognition, with males showing worse performances on verbal memory and executive tests (Abraham et al. 2023) but better visuospatial abilities (Liu et al. 2015).

In contrast, females tend to perform better than males in verbal abilities, including semantic and phonemic verbal fluency (Szewczyk‐Krolikowski et al. 2014), as well as in long‐term verbal memory, measured by both immediate and delayed recall (Bayram et al. 2020). Finally, notable sex differences have been observed in social cognition (Clark et al. 2008), with males generally being less accurate than females in identifying fearful expressions.

However, it must be acknowledged that these reported differences are well‐known even in the healthy population (Levy and Heller 1992), and it is therefore unclear whether, in the case of PD patients, they depend on specific pathological mechanisms differently affecting males and females.

The literature focused on sex differences in neuropsychiatric symptoms reports more frequently irritability, sadness, and lack of motivation in PD females compared to their male counterparts (Meoni et al. 2020), as well as anxiety and depression (Szewczyk‐Krolikowski et al. 2014; Solla et al. 2012; Song et al. 2014). These findings are in line with what is observed in the general population, in which anxiety and depression are more frequently reported in females than in males (Rubinow and Schmidt 2018).

Furthermore, the characterization of sex‐related cognitive and behavioral differences in PD is complicated by the presence of different motor phenotypes (Jankovic et al. 1990), which may also affect performance. Individuals with a TD phenotype have been described as less subjected to cognitive decline compared to those with symptoms such as rigidity (RA phenotype) (Jankovic and Kapadia 2001), performing better in executive functions, such as working memory, phonemic word fluency, attention, and visuospatial abilities (Wojtala et al. 2019). While the TD phenotype tends to be more frequent than the RA one in females and is associated with a more benign onset (Haaxma et al. 2007; Solla et al. 2012; Picillo et al. 2017), it is still unclear whether the sex‐related differences in cognitive performance can be traced back to the different distribution of motor phenotypes, as no study has yet investigated the interaction between sex and the type of motor symptoms specifically at onset.

Another variable that seems to affect PD evolution is lateralization at onset, with a left onset having a more favorable progression than a right one (Varoğlu et al. 2021). In addition, patients with left‐sided onset usually are more impaired in visuospatial memory tasks, while those with right onset typically perform worse on verbal memory tasks with spared orientation and mental imagery tests (Verreyt et al. 2011).

While the effects on cognitive functioning of sex and lateralization at onset have been investigated separately in PD, the possible interaction between these factors is still under debate, with both positive (Davidsdottir et al. 2008) and negative findings (Bentivoglio et al. 2023). More specifically, preliminary evidence on a line bisection test showed in male patients with left‐side onset a rightward deviation on the egocentric midline compared to females with the same lateralization, who were close to the true midpoint (Davidsdottir et al. 2008). In contrast, no interaction effects were found by Bentivoglio et al. (2023) on any of the performed tasks.

To the best of our knowledge, no studies have investigated the possible interaction between sex, motor phenotype, and lateralization at onset on cognitive and behavioral functioning.

Therefore, this study aims to analyze in a large group of PD patients the effects of sex and motor features at onset (lateralization of symptoms and motor phenotype) on cognitive and neurobehavioral functioning, under the hypothesis that combining these factors might lead to different clinical phenotypes. Given that the TD phenotype appears more frequent in females (Haaxma et al. 2007; Solla et al. 2012) and is associated with lower cognitive impairment (Wojtala et al. 2019), we hypothesized that TD females would generally perform better on cognitive tasks.

## 2 Materials and Methods

### 2.1 Participants

In this retrospective and cross‐sectional study, data from 304 patients with idiopathic PD (119 women and 185 men) were considered. All participants had a diagnosis of PD (Postuma et al. 2015), a Hoehn and Yahr score ≤ 3, and were native Italian speakers. Exclusion criteria were a diagnosis of atypical Parkinsonism, a diagnosis of PD–dementia (Emre et al. 2007), a history of advanced therapy, the presence of confirmed genetic mutations related to PD, or any other medical condition that could affect cognitive functioning (Longo et al. 2024).

Patients were referred to the Neurocognitive Rehabilitation Center of Rovereto (CeRiN), Italy. Neurologists collected data on the patient's age and motor lateralization at onset, disease duration, Hoehn and Yahr score, and motor phenotype (TD or RA) (Moretti et al. 2017). Each enrolled patient underwent a comprehensive neuropsychological and neurobehavioral assessment, administered by an expert clinical neuropsychologist. All patients gave their informed consent prior to their inclusion in this study. The study was approved by the local ethical committee (University of Trento Research Ethics Committee, protocol 2014‐010).

### 2.2 Neuropsychological Assessment

An in‐depth neuropsychological evaluation was performed by using standardized versions currently used for the Italian population, with available correction for significant demographic variables (i.e., age, education, and sex, when necessary). Although we recognize that the neuropsychological tests are multifactorial in nature (e.g., verbal fluency tasks assessing both language and executive functions), tasks were selected to evaluate all the core cognitive domains based on the DSM‐5 guidelines (Sachdev et al. 2014). In particular, verbal short‐term memory and working memory were assessed using the Digit Span (Monaco et al. 2013), while nonverbal short‐term memory was assessed with the Corsi span forward (Monaco et al. 2013). Attention was evaluated using the attentional matrices (Spinnler and Tognoni et al. 1987), verbal learning and long‐term memory through the Rey Auditory Verbal Learning Test (RAVLT) (Carlesimo et al. 1996), while nonverbal long‐term memory by means of the delayed recall of the Rey–Osterrieth Complex Figure (ROCF) (Caffarra et al. 2002). The copy of this figure was used to assess visuo‐constructive abilities; visuospatial, and visuo‐perceptual abilities were assessed using the Line Orientation Judgment (Benton et al. 2012) and the Unknown Face Recognition Test (Benton et al. 1978). Executive functions were evaluated using the Stroop Color Word Test (SCWT), computing both accuracy and reading speed interference indexes (Caffarra et al. 2024), the Trail Making Test Parts A and B (Giovagnoli et al. 1996) for divided attention, and the phonemic verbal fluency task for cognitive flexibility. Language was assessed using the object (Catricalà et al. 2013) and action (Papagno et al. 2020) naming tests and the semantic verbal fluency task (Zarino et al. 2014). Finally, social cognition was evaluated using the Ekman 60‐Faces test (Dodich et al. 2014).

Patients also underwent a series of functional and neurobehavioral questionnaires, namely the Geriatric Depression Scale, a 30‐item self‐administered questionnaire assessing depression symptoms in geriatric subjects over the previous week (Yesavage et al. 1982), and the Parkinson Anxiety Scale, a 12‐item scale to evaluate anxiety specifically in PD patients (Leentjens et al. 2014). The neuropsychological evaluation required overall 2 hours and was performed in one or two different sessions based on the patients’ availability, who were tested ON medication.

### 2.3 Statistical Analysis

Statistical analyses were performed to evaluate the effect of sex, motor phenotype, and lateralization at onset on cognitive performance. We first conducted preliminary independent sample *t*‐tests to verify whether male and female groups showed any difference in demographic variables (i.e., age, education, and disease duration). Parametric or nonparametric statistics were used based on data distribution. We also performed chi‐squared tests to assess significant differences in the distribution due to onset phenotype and onset lateralization.

Given the large number of variables, a preliminary Principal Component Analysis (PCA) was conducted on raw scores from neuropsychological tests and behavioral questionnaires to reduce dimensionality by extracting a smaller number of components. Hereafter, for each domain obtained, a 2 × 2 × 2 multivariate analysis of covariance (MANCOVA) was conducted, with sex (female vs. male), motor phenotype at onset (RA phenotype vs. TD phenotype), and lateralization of motor symptoms at onset (right‐onset vs. left‐onset) as independent variables. Age and years of education were included as covariates. When significant *p* values were found in the multivariate tests, univariate follow‐up analyses were then performed to identify group differences. Corrections for multiple comparisons (false discovery rate) were also applied; significant adjusted *p* values are reported alongside the MANCOVA values throughout the text. To control for possible statistical differences driven by the different numerosity of the subgroups, exploratory MANCOVAs were also performed considering data from 200 patients, 50 for each group, which were selected randomly after removing eventual outliers. However, to rule out that the differences in male–female performance were due to the “physiological” differences described in the literature (e.g., better performance in emotion recognition in females; better performance in visuospatial skills in males), a control ANOVA analysis was performed using the adjusted (for age, education, and sex) scores. These scores are computed based on Capitani and Laiacona's (1988) procedure and allow us to consider the effect of relevant demographic variables (e.g., age, education, and sex) on task performance.

This procedure is a regression‐based statistical method widely used in Italian clinical neuropsychology. More in detail, raw scores are adjusted for age, education, and, when indicated, for sex, according to the parameters estimated in a normal sample (usually between 200 and 350 neurologically unimpaired subjects) with a multiple regression model. Adjusted scores of 5% one‐sided nonparametric tolerance limit (with 95% CI) are considered pathological: inferential cut‐off scores are therefore those at which or below which the probability that an individual belongs to the normal population is < 0.05 (Capitani and Laiacona 2017).

A significant difference between PD males and females using adjusted scores indicates that the results do not depend on sex per se but rather suggest a specific sex effect related to the disease.

For statistical analysis, Jamovi was used (https://www.jamovi.org; version 2.6.13). The level of significance was set at *p* < 0.05.

## 3 Results

Male and female groups showed no difference in demographic variables apart from education (*p* = 0.003), with a higher level of education in males. Significant differences were found in onset phenotype distribution (*p* = 0.026), with a higher percentage of RA onset in males compared to females (Table 1). No significant differences were found, instead taking into consideration onset lateralization.

**TABLE 1:** Demographic and general clinical characteristics of PD patients, divided by sex, onset motor phenotype, and onset motor lateralization.

	Sex		
	Females	Males	*p*
Demographic features			
*N* (percentage)	119 (39.1)	185 (60.9)	
Age, y (mean ± SD)	66.45 ± 9.33	68.2 ± 7.32	0.078^a^
Education, y (mean ± SD)	9.61 ± 3.14	10.9 ± 4.26	**0.003** ^a^
Disease duration, m (mean ± SD)	68.02 ± 57.68	75.0 ± 56.24	0.128^b^
Motor assessment			
Onset phenotype			**0.026** ^c^
RA onset (percentage)	50 (16.4)	102 (33.6)	
TD onset (percentage)	69 (22.7)	83 (27.3)	
Onset lateralization			0.826^c^
Right‐side onset (percentage)	64 (21.3)	95 (31.7)	
Left‐side onset (percentage)	55 (18.3)	86 (28.7)	
	**Onset motor phenotype**		
	**Rigid‐akinetic onset**	**Tremor‐dominant onset**	***p***
Demographic features			
*N* (percentage)	152 (50.0)	152 (50.0)	
Age, y (mean ± SD)	67.1 ± 8.19	67.99 ± 78.21	0.326^b^
Education, y (mean ± SD)	10.8 ± 4.05	9.97 ± 3.71	0.078^b^
Disease duration, m (mean ± SD)	73.3 ± 54.73	71.26 ± 58.99	0.468^b^
	**Onset motor lateralization**		
	**Right‐side onset**	**Left‐side onset**	***p***
Demographic features			
*N* (percentage)	159 (53.0)	141 (47.0)	
Age, y (mean ± SD)	67.0 ± 8.09	68.2 ± 8.36	0.135^b^
Education, y (mean ± SD)	10.3 ± 3.92	10.4 ± 3.89	0.741^b^
Disease duration, m (mean ± SD)	72.7 ± 54.27	69.5 ± 56.44	0.553^b^

*Note*: Significant *p* values are reported in bold.

Abbreviations: m, month; *N*, number; *p*, *p* value; PD, Parkinson's disease; RA, rigid‐akinetic onset phenotype; SD, standard deviation;

TD, tremor‐dominant onset phenotype.

^a^ Welch's test.

^b^ Mann–Whitney test.

^c^ *χ* ^2^ test.

Conducting preliminary independent sample *t*‐tests on the convenience sample, males and females did not significantly differ in any demographic and clinical variables (Table 2).

**TABLE 2:** Demographic and general clinical characteristics of PD patients in the convenience sample, divided by sex.

	Females	Males	*p*
Demographic features			
*N* (percentage)	100 (50.0)	100 (50.0)	
Age, y (mean ± SD)	66.95 ± 8.98	68.04 ± 7.37	0.355^a^
Education, y (mean ± SD)	9.80 ± 3.04	9.92 ± 3.59	0.887^a^
Disease duration, m (mean ± SD)	69.25 ± 56.42	73.60 ± 55.93	0.414^a^
Motor assessment			
Onset phenotype			1.00^b^
RA onset (percentage)	50 (25.0)	50 (25.0)	
TD onset (percentage)	50 (25.0)	50 (25.0)	

*Note*: Significant *p* values are reported in bold.

Abbreviations: m, month; N, number; *p*, *p* value; PD, Parkinson's disease; RA, rigid‐akinetic onset phenotype; SD, standard deviation; TD, tremor‐dominant onset phenotype; y, years.

^a^ Mann–Whitney test.

^b^ *χ* ^2^ test.

The PCA performed on neuropsychological and behavioral scores identified four components (cumulative total variance 55.4%). Related tasks were grouped together to reflect different functional domains: the executive‐attentional domain (i.e., Trail Making Part A, Trail Making Test Part B, Trail Making Test Part (B–A), Attentional Matrices, SWCT—Time Interference, Digit span forward task, Digit span backward task, Corsi block forward task), the memory and visuospatial domain (i.e., RAVLT—Immediate Recall, RAVLT—Delayed Recall, ROCF—Copy, ROCF—Delayed Recall, SWCT—Error Interference, Unknown Face Recognition Test, Line Orientation Judgment Test), the lexical‐semantic domain (Object naming test, Action naming test, Semantic fluency test, Phonemic fluency test, Ekman‐60 Faces Global Score), and behavioral domain (Geriatric Depression Scale, Parkinson Anxiety Scale), assessing self‐reported depression and anxiety symptoms (Table 3).

**TABLE 3:** Principal Component Analysis results of neuropsychological and behavioral measures, divided by cognitive domain.

	Component			
	Executive and attentional	Memory and visuospatial	Semantic	Behavioral
Trail Making Test Part B	−0.809	−0.331		
Attentional matrices	0.795			
Trail Making Test Part A	−0.755			
Trail Making Test Part (B–A)	−0.722	−0.313		
SCWT—time interference	−0.589	−0.324		
Digit span forward task	0.519		0.415	
Digit span backward task	0.503		0.412	
Corsi block forward task	0.455	0.388		
RAVLT—delayed recall		0.636		
ROCF—delayed recall		0.604		
SCWT—error interference		−0.589		
ROCF—copy		0.587		
RAVLT—immediate recall	0.365	0.575		
Face Recognition Test		0.531		
Judgment Line Orientation Test		0.387	0.321	
Object naming test			0.809	
Action naming test			0.783	
Phonemic fluency test	0.488		0.526	
Ekman‐60 Faces Global score	0.313	0.399	0.500	
Semantic fluency test	0.465	0.319	0.487	
Geriatric Depression Scale				0.852
Parkinson Anxiety Scale				0.851

Abbreviations: RAVLT, Rey Auditory Verbal Learning Test; ROCF, Rey–Osterrieth Complex Figure; SCWT, Stroop Color Word Test.

Preliminary data analysis to verify MANCOVA assumptions detected violations in terms of homogeneity of covariance (Box's *M* test: *p* < 0.001) and multivariate normality (*p* < 0.001) in all analyses apart from the MANCOVA evaluating mood disorders (Box's *M* test: *χ* ^2^ (21) = 21.8, *p* = 0.41). As the MANCOVA represents a robust statistical method (Ateş et al. 2019), particularly when the sample sizes are large and groups are approximately equal in size, the analyses were continued despite these violations, using Pillai's Trace (*V*) as recommended (Ateş et al. 2019). However, the results should be interpreted cautiously due to possible inflated Type I error rates.

The MANCOVA conducted on executive‐attentional measures (Table 4) showed no significant effects of sex (*F* (8, 248) = 1.398, *p* = 0.19761), motor phenotype at onset (*F* (8, 248) = 1.469, *p* = 0.1691), or motor lateralization at onset (*F* (8, 248) = 0.887, *p* = 0.52816), nor any significant interactions between these three factors.

**TABLE 4:** MANCOVA's results of executive and attentional measures.

Multivariate tests					
	Pillai's trace	*F*	df1	df2	*p*
Sex	0.0432	1.398	8	248	0.19761
Onset phenotype	0.0452	1.469	8	248	0.16910
Onset lateralization	0.0278	0.887	8	248	0.52816
Sex × onset phenotype	0.0505	1.650	8	248	0.11138
Sex × onset lateralization	0.0373	1.200	8	248	0.29941
Onset phenotype × onset lateralization	0.0265	0.843	8	248	0.56528
Sex × onset phenotype × onset lateralization	0.0486	1.584	8	248	0.13005

Abbreviations: df, degree of freedom; *F*, *F*‐statistic; *p*, *p* value.

Similarly, in the smaller sample, the MANCOVA on executive‐attentional measures did not find any significant effects (Table 5).

**TABLE 5:** MANCOVA's results of executive and attentional measures of 200 patients.

Multivariate tests					
	Pillai's trace	*F*	df1	df2	*p*
Sex	0.0713	1.477	8	154	0.16963
Onset phenotype	0.0781	1.632	8	154	0.11996
Onset lateralization	0.0420	0.844	8	154	0.56551
Sex × onset phenotype	0.0741	1.541	8	154	0.14727
Sex × onset lateralization	0.0666	1.373	8	154	0.21232
Onset phenotype × onset lateralization	0.0391	0.784	8	154	0.61771
Sex × onset phenotype × onset lateralization	0.0863	1.817	8	154	0.07771

Abbreviations: df, degree of freedom; *F*, *F*‐statistic; *p*, *p* value.

Considering memory and visuospatial measures, the MANCOVA (Table 6) revealed a significant effect of sex (*F*(7,229) = 9.252, *p* < 0.0001, FDR‐corrected *p* = 0.00028). Follow‐up univariate analyses showed that female patients reported higher scores than their male counterparts in both the immediate (*F*(1,235) = 14.7052, *p* = 0.00016) and the delayed recall (*F*(1, 235) = 17.84181, *p* = 0.00003) of RAVLT, while males performed significantly better in the Line Orientation Judgment task (*F*(1, 235) = 28.22553, *p* < 0.00001). A control analysis, performed using the adjusted scores, did not confirm a statistically significant difference between male and female patients’ scores in the Line Orientation Judgment task (*F*(1, 267) = 3.415, *p* = 0.06570). A tendency to significance was found in the interaction between sex and onset phenotype (*F*(7, 229) = 1.880, *p* = 0.07378).

**TABLE 6:** MANCOVA's results of memory and visuospatial measures.

Multivariate tests					
	Pillai's trace	*F*	df1	df2	*p*
Sex	0.2205	9.252	7	229	**< 0.00001**
Onset phenotype	0.0364	1.234	7	229	0.28489
Onset lateralization	0.0346	1.173	7	229	0.31938
Sex × onset phenotype	0.0543	1.880	7	229	0.07378
Sex × onset lateralization	0.0267	0.897	7	229	0.50962
Onset phenotype × onset lateralization	0.0145	0.482	7	229	0.84685
Sex × onset phenotype × onset lateralization	0.0281	0.944	7	229	0.47316
**Univariate Tests**					
Sex		***F***	**df**		***p***
RAVLT—immediate recall		14.7052	1		**0.00016**
RAVLT—delayed recall		17.8418	1		**0.00003**
ROCF—copy		0.01803	1		0.89329
ROCF—delayed recall		48.1333	1		0.20052
SWCT—error interference		0.85607	1		0.35579
Unknown Face Recognition Test		0.18661	1		0.66615
Line Orientation Judgment Test		28.2255	1		**< 0.00001**

*Note*: Significant *p* values are reported in bold.

Abbreviations: df, degree of freedom; *F*, *F*‐statistic; *p*, *p* value; RAVLT, Rey Auditory Verbal Learning Test; ROCF, Rey–Osterrieth Complex Figure; SCWT, Stroop Color Word Test.

The MANCOVA conducted on the 200 patients (Table 7) revealed a significant main effect of sex (*F* (7, 145) = 8.111, *p* < 0.00001, FDR‐corrected *p* = 0.00028). As in the whole sample, female patients outperformed their male counterparts in both the immediate (*F* (1, 151) = 24.63114, *p* < 0.00001) and the delayed recall (*F* (1, 151) = 18.65586, *p* = 0.00003) of RAVLT, while males performed better in the Line Orientation Judgment (*F* (1, 151) = 12.02501, *p* = 0.0068). An additional ANOVA performed on adjusted scores did not reveal any significant difference between males’ and females’ performances in the Line Orientation Judgment task (*F* (1, 171) = 3.104, *p* = 0.080). A significant interaction between sex and phenotype was also found at the multivariate level (*F* (7, 145) = 0.01830, *p* = 0.01830), reflecting differences in the immediate recall of RAVLT (*F* (1, 151) = 6.98003, *p* = 0.00911), in the SCWT Error Interference index (*F*(1, 151) = 4.35738, *p* = 0.03853), and in the Face Recognition Test (*F* (1, 151) = 10.20313, *p* = 0.00171). RA females performed better than the other groups in both the immediate recall of RAVLT and the Unknown Face Recognition task, while TD men outperformed the other patients in the SCWT Error Interference index. TD males showed the worst performance in the immediate recall of RAVLT, while RA males performed at the worst level in the Unknown Face Recognition task compared to the other groups. Moreover, TD females reported the highest average number of errors in the SCWT Error Interference index compared to the other patients.

**TABLE 7:** MANCOVA's results of memory and visuospatial measures of 200 patients.

Multivariate tests					
	Pillai's trace	*F*	df1	df2	*p*
Sex	0.2814	8.111	7	145	**< 0.00001**
Onset phenotype	0.0587	1.292	7	145	0.25811
Onset lateralization	0.0467	1.014	7	145	0.42385
Sex × onset phenotype	0.1081	2.510	7	145	**0.01830**
Sex × onset lateralization	0.0456	0.989	7	145	0.44188
Onset phenotype × onset lateralization	0.0504	1.099	7	145	0.36681
Sex × onset phenotype × onset lateralization	0.0604	1.332	7	145	0.23937
**Univariate Tests**					
Sex		***F***	**df**		***p***
RAVLT—immediate recall		24.6311	1		**< 0.00001**
RAVLT—delayed recall		18.6559	1		**0.00003**
ROCF—copy		0.10381	1		0.74775
ROCF—delayed recall		1.01778	1		0.31466
SWCT—error interference		0.02089	1		0.88527
Unknown Face Recognition Test		1.15842	1		0.28351
Line Orientation Judgment Test		12.0250	1		**0.00068**
Sex × Onset phenotype					
RAVLT—immediate recall		6.98003	1		**0.00911**
RAVLT—delayed recall		1.32757	1		0.25106
ROCF—copy		2.88447	1		0.09150
ROCF—delayed recall		3.16051	1		0.07745
SWCT—error interference		4.35738	1		**0.03853**
Unknown Face Recognition Test		10.2031	1		0.27848
Line Orientation Judgment Test		1.18298	1		**0.00171**

*Note*: Significant *p* values are reported in bold.

Abbreviations: df, degree of freedom; *F*, *F*‐statistic; *p*, *p* value; RAVLT, Rey Auditory Verbal Learning Test; ROCF, Rey–Osterrieth Complex Figure; SCWT, Stroop Color Word Test.

In the semantic domain, the MANCOVA (Table 8) showed a significant effect of sex (*F* (5, 239) = 9.472, *p* < 0.00001, FDR‐corrected *p* = 0.00014). Specifically, females outperformed males in the Object Naming Test (*F*(1, 243) = 4.42913, *p* = 0.03636). but males reported higher scores in semantic fluency (F (1, 243_ 4.19032, p = 0.04173). Females also outperformed males in the Ekman‐60 Faces Global Score (*F* (1.243) = 14.76026, *p* = 0.00016). The control ANOVA, performed on adjusted Ekman‐60 Faces global scores, confirmed that female patients scored significantly higher in emotion recognition than male patients (*F* (1, 263) = 12.95994, *p* = 0.00038).

**TABLE 8:** MANCOVA's results of semantic measures.

Multivariate tests					
	Pillai's trace	*F*	df1	df2	*p*
Sex	0.1654	9.472	5	239	**< 0.00001**
Onset phenotype	0.0156	0.756	5	239	0.58239
Onset lateralization	0.0378	1.878	5	239	0.09886
Sex × onset phenotype	0.0335	1.657	5	239	0.14581
Sex × onset lateralization	0.0136	0.661	5	239	0.65326
Onset phenotype × onset lateralization	0.0181	0.881	5	239	0.49416
Sex × onset phenotype × onset lateralization	0.0190	0.923	5	239	0.46641
**Univariate Tests**					
Sex		***F***	**df**		***p***
Object naming test		4.42913	1		**0.03636**
Action naming test		1.43551	1		0.23203
Semantic fluency test		4.19032	1		**0.04173**
Phonemic fluency test		0.01471	1		0.90355
Ekman‐60 Faces Global Score		14.7603	1		**0.00016**

*Note*: Significant *p* values are reported in bold.

Abbreviations: df, degree of freedom; *F*, *F* statistic; *p*, *p* value.

Considering the smaller sample (Table 9), the MANCOVA revealed again a significant effect of sex (*F* (5, 151) = 7.674, *p* < 0.00001, FDR‐corrected *p* = 0.00014). At the univariate level, females performed significantly better in the Object Naming test (*F*(1, 155) = 4.79161, *p* = 0.03009) and in the Ekman 60‐Faces Global Score (*F* (1,155) = 14.69602, *p* = 0.00018). Again, the control ANOVA on the adjusted scores confirmed that women outperformed men in the Ekman‐60 Faces Global Score (*F* (1, 166) = 15.990, *p* = 0.0001).

**TABLE 9:** MANCOVA's results of the semantic measures of 200 patients.

Multivariate tests					
	Pillai's trace	*F*	df1	df2	*p*
Sex	0.2026	7.674	5	151	**< 0.00001**
Onset phenotype	0.0187	0.574	5	151	0.71985
Onset lateralization	0.0628	2.024	5	151	0.07841
Sex × onset phenotype	0.0556	1.778	5	151	0.12073
Sex × onset lateralization	0.0254	0.788	5	151	0.55980
Onset phenotype × onset lateralization	0.0292	0.623	5	151	0.68243
Sex × onset phenotype × onset lateralization	0.0536	1.712	5	151	0.13523
**Univariate Tests**					
Sex		***F***	**df**		***p***
Object naming test		4.79161	1		**0.03009**
Action naming test		0.07648	1		0.78250
Semantic fluency test		1.74816	1		0.18806
Phonemic fluency test		1.96005	1		0.16351
Ekman‐60 Faces Global Score		14.6960	1		**0.00018**

*Note*: Significant *p* values are reported in bold.

Abbreviations: df, degree of freedom; *F*, *F*‐statistic; *p*, *p* value.

Finally, multivariate tests on behavioral questionnaires (Table 10) showed a significant main effect of sex (*F*(2, 259) = 10.0718, *p* = 0.00006, FDR‐corrected *p* = 0.00056). Univariate analyses revealed that female patients reported higher levels in both the Geriatric Depression Scale (*F* (1, 260) = 18.2161, *p* = 0.00003) and the Parkinson Anxiety Scale (*F*(1, 260) = 13.8474, *p* = 0.00024).

**TABLE 10:** MANCOVA's results of behavioral measures.

Multivariate tests					
	Pillai's trace	*F*	df1	df2	*p*
Sex	0.0722	10.07	2	259	**0.00006**
Onset phenotype	0.0006	0.080	2	259	0.92314
Onset lateralization	0.0186	2.460	2	259	0.08742
Sex × Onset phenotype	0.0216	2.862	2	259	0.05896
Sex × Onset lateralization	0.0112	1.460	2	259	0.23419
Onset phenotype × Onset lateralization	0.0029	0.381	2	259	0.68327
Sex × Onset phenotype × Onset lateralization	0.0033	0.574	2	259	0.56404
**Univariate Tests**					
Sex		***F***	**df**		***p***
Geriatric Depression Scale		18.216	1		**0.00003**
Parkinson Anxiety Scale		13.847	1		**0.00024**

*Note*: Significant *p* values are reported in bold.

Abbreviations: df, degree of freedom; *F*, *F*‐statistic; *p*, *p* value.

**FIGURE 1:**
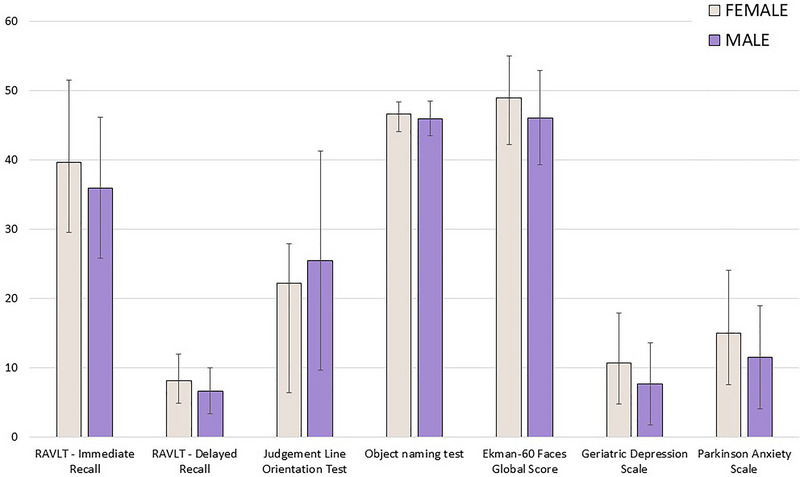
Significant differences in sex analyses for cognitive and behavioral variables. RAVLT, Rey Auditory Verbal Learning Task.

**FIGURE 2:**
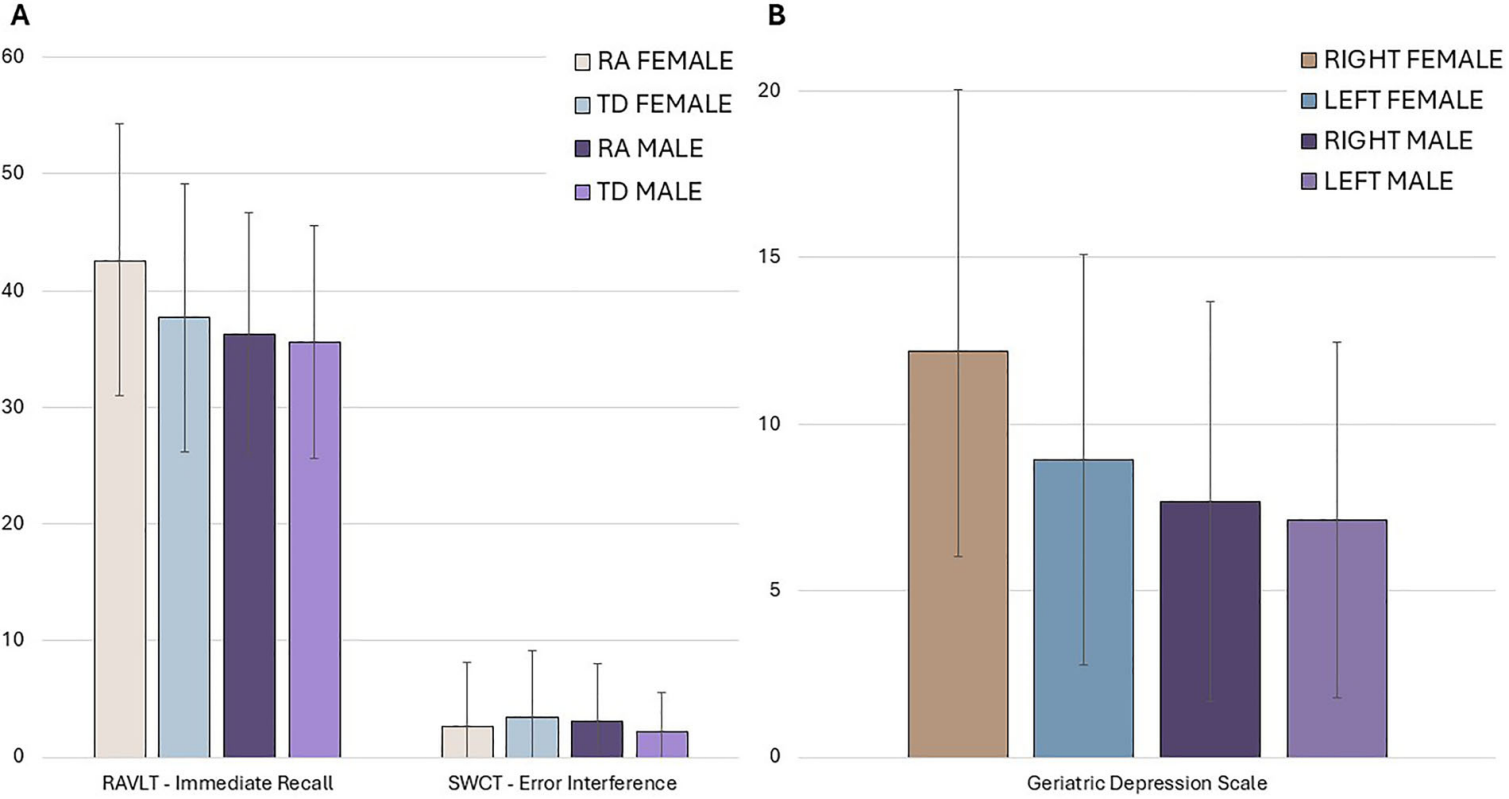
Significant differences in sex x motor phenotype (A) and sex × motor lateralization (B) analyses considering the convenience sample of 200 patients. RA, rigid‐akinetic phenotype; RAVLT, Rey Auditory Verbal Learning Test; SWCT, Stroop Color Word Test; TD, tremor‐dominant phenotype;

The MANCOVA on behavioral questionnaires (Table 11) in the convenience sample found a significant effect of sex (*F* (2, 168) = 10.588, *p* = 0.00005, FDR‐corrected *p* = 0.000467). Univariate follow‐up analyses confirmed that females self‐reported higher levels of both depression (*F* (1, 169) = 15.384, *p* = 0.00013) and anxiety (*F* (1, 169) = 18.991, *p* = 0.00002). In addition, a significant effect of the interaction between sex and onset motor lateralization was found for depression (*F*(1, 169) = 6.893, *p* = 0.00945), with females with right‐side onset reporting the highest levels of depressive symptoms, whereas males with the same lateralization reported the lowest. See Figures 1 and 2 for a graphical representation of the results of analyses on cognitive and behavioral variables.

**TABLE 11:** MANCOVA's results of behavioral measures of 200 subjects.

Multivariate tests					
	Pillai's trace	*F*	df1	df2	*p*
Sex	0.1119	10.59	2	168	**0.00005**
Onset phenotype	0.0288	2.491	2	168	0.08587
Onset lateralization	0.0163	1.389	2	168	0.25221
Sex × Onset phenotype	0.0249	2.143	2	168	0.12052
Sex × Onset lateralization	0.0392	3.430	2	168	**0.03467**
Onset phenotype × Onset lateralization	0.0241	2.076	2	168	0.12859
Sex × Onset phenotype × Onset lateralization	0.0023	0.191	2	168	0.82662
**Univariate Tests**					
Sex		***F***	**df**		***p***
Geriatric Depression Scale		15.384	1		**0.00013**
Parkinson Anxiety Scale		18.991	1		**0.00002**
Sex × Onset lateralization					
Geriatric Depression Scale			6.8930	1	**0.00945**
Parkinson Anxiety Scale			2.5010	1	0.11564

*Note*: Significant *p* values are reported in bold.

Abbreviations: df, degree of freedom; *F*, *F*‐statistic; *p*, *p* value.

**FIGURE 3:**
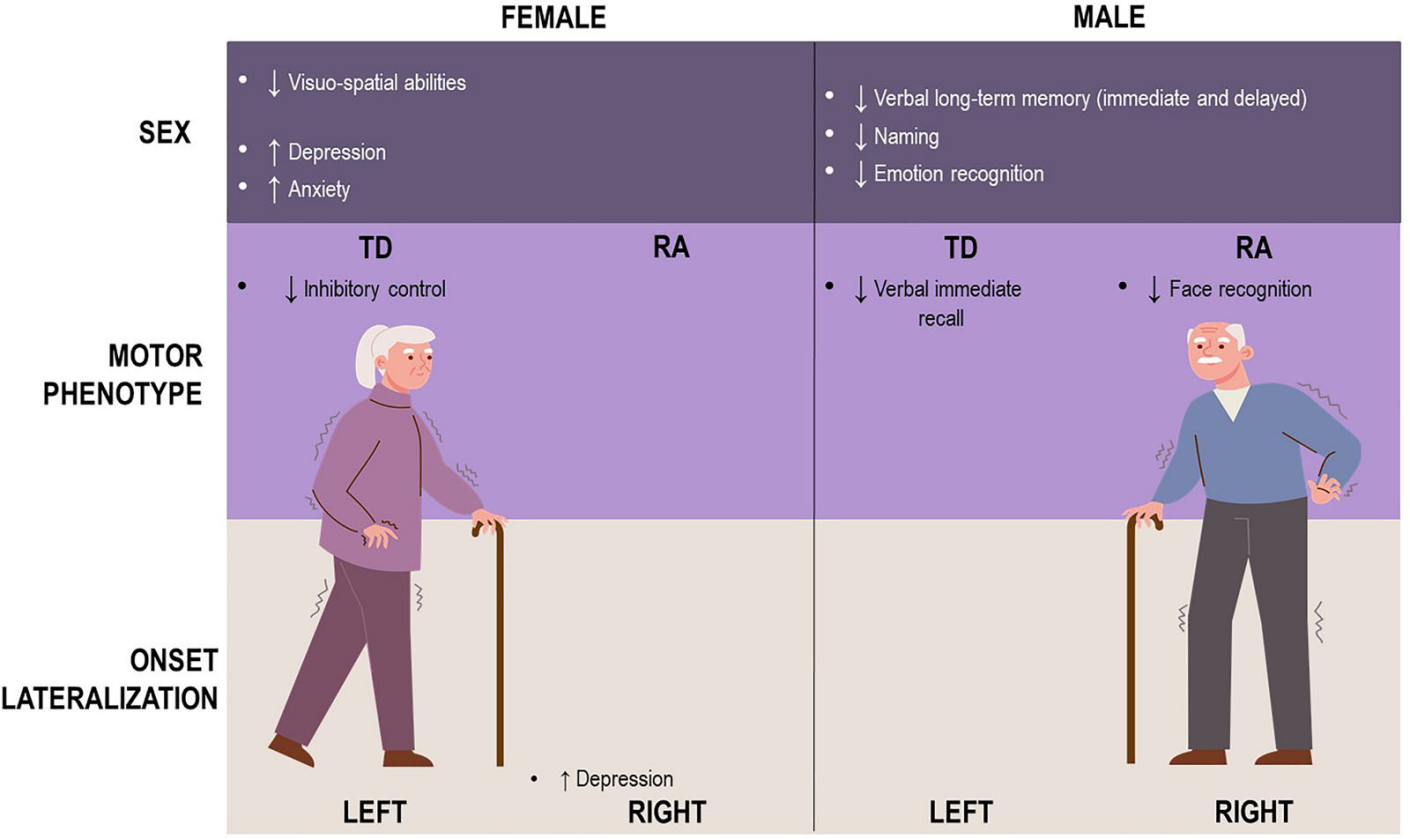
Summary of cognitive and behavioral differences found between female and male PD patients. RA, rigid‐akinetic; TD, tremor dominant.

For a summary of cognitive and behavioral differences, see Figure 3.

## 4 Discussion

Sex differences in PD clinical profiles have been previously reported. Although still not fully elucidated, the reason for these differences is likely multifactorial (Heller et al. 2013), as sex influences the normal functioning of nigrostriatal dopaminergic pathways (Gillies et al. 2014), individuals’ lifestyles, and the pathogenetic mechanisms and progression in PD (Smith and Dahodwala 2014). On this background, there is a strong need for a better understanding of brain sex dimorphism in this neurodegenerative condition for its potential translational impact on patients' monitoring and definition of a personalized path of care.

To our knowledge, this is the first study to assess sex differences in the neuropsychological profiles of PD patients, taking into consideration also onset phenotype and symptom lateralization.

Overall, the present findings show significant differences between males and females in terms of cognitive performance and neurobehavioral symptoms. In accordance with previous literature (Liu et al. 2015; Szewczyk‐Krolikowski et al. 2014; Bayram et al. 2020; Solla et al. 2012; Song et al. 2014), females showed better performance in verbal episodic memory but also higher levels of mood disturbances (i.e., anxiety and depression). These results were consistent when analyzing both the full sample and the convenience sample. Our results also extend this advantage to other cognitive domains, such as attention and naming.

In addition, sex differences emerged in the visuospatial and semantic domains. In particular, males and females also differed in emotion recognition (i.e., lower performance in males) and visuospatial abilities (i.e., lower performance in females). These findings have been described previously in healthy populations (Clark et al. 2008; Levy and Heller 1992), thus making it difficult to distinguish whether the results reported on PD simply reflect these sex differences or whether there are additional sex‐related mechanisms that come into play specifically in the PD course. Since no significant differences were found in the case of visuospatial abilities when using adjusted scores, our findings support the first hypothesis. On the other hand, reduced emotion recognition in males was confirmed, suggesting a vulnerability in this cognitive function in male PD patients. Although the causes of this imbalance in social cognition performance are still to be fully elucidated, it is possible to speculate that it might be at least partially related to more severe structural neurodegeneration in PD males in regions involved in emotion processing (Yadav et al. 2016).

Another possible explanation might consider previous literature showing higher accuracy for recognizing negative emotions in females with depressive disorders (Biyik et al. 2015), although no clear evidence in PD patients has been found (Pietschnig et al. 2016).

However, in our sample, no significant correlation was found between emotion recognition (i.e., the Ekman 60‐Faces test) and mood disorders in female PD individuals (Geriatric Depression Scale: *r* = −0.122, *p* = 0.23; Parkinson's Anxiety Scale: *r* = −0.003, *p* = 0.97); thus, this current hypothesis does not seem likely.

Previous findings on cohort studies highlighted more pronounced structural changes in male rather than female patients (Beheshti et al. 2024; Oltra et al. 2024), including frontal, parietal, or limbic regions, that were associated with increased cognitive decline. Interestingly, PD females showed structural vulnerabilities limited to temporoparietal brain regions (Oltra et al. 2024), which is in line with our behavioral findings.

Furthermore, sex hormones might play a relevant role in PD differences between males and females, as estrogens have been found to have neuroprotective effects on the nigrostriatal dopaminergic system and a modulating role on monoamine oxidase (Jurado‐Coronel et al. 2018). It has been recently suggested that whereas estradiol might have a protective role in motor impairment, testosterone might be involved in male vulnerability to PD neuropathology (Bovenzi et al. 2023). In addition to previous literature, the significant interaction between onset motor phenotype and sex represents another relevant finding of the current study. In particular, females with the RA phenotype at onset performed better compared to the other groups in verbal memory and visuoperceptive tasks, while TD males showed the worst performance on a verbal learning task. In contrast, RA males reported the lowest scores in unknown face recognition, and TD females made more errors in an executive interference task. While previous evidence supports the relationship between RA phenotype and cognitive dysfunctions (Alves et al. 2006), these findings suggest that the combination of sex and motor phenotype might exacerbate the extent of cognitive deterioration. To the best of our knowledge, there are no specific studies investigating in‐depth differences between males and females within the RA phenotype, although preliminary evidence suggests that rigidity might have a different weight in PD diagnosis in males versus females (Angelini et al. 2024). One possible explanation might rely on the overlap between the neural networks affected in this specific motor phenotype and those more vulnerable in males. In fact, previous findings showed that akinetic‐rigid symptoms are particularly associated with the decline in frontoparietal networks involved in motor and executive control (Kann et al. 2020). On the other hand, PD males show higher structural alterations compared to females in both frontal and parietal regions (Oltra et al. 2024). Despite future studies are needed to understand the combined effects of these two factors, it is possible to speculate that the combination of the two might exacerbate the clinical picture due to the synergic negative effects on the same systems/networks. Another possible explanation might call into play the differential vulnerability of the nigrostriatal dopaminergic system in males compared to females, involving brain structures implicated in both motor (rigidity) and non‐motor (executive functions) symptoms (Vegeto et al. 2020).

Future studies should define the nature of the interaction between these factors and the underlying mechanisms. An extremely interesting result was, however, that RA females performed better on verbal learning than TD males, suggesting that sex effect overcomes motor phenotype in the case of this cognitive function.

Whether these differences might be explained in terms of differential circuits involved in the two motor phenotypes (Wojtala et al. 2019). or considering the higher susceptibility of females to mood disorders in general (Rubinow and Schmidt 2018), is still to be elucidated.

Finally, consistently with previous literature (Bentivoglio et al. 2023), no interaction between sex and symptom lateralization was found in the cognitive domain. However, results from neurobehavioral questionnaires revealed that patients with right‐onset of PD, particularly female patients, self‐report more depressive symptoms compared to those with left‐onset. Although the relationship between mood disorders and lateralization requires further studies to be fully clarified, it is possible to speculate that this finding might be at least partially linked to the worst prognosis reported in the literature for right‐onset patients (Varoğlu et al. 2021), as depressive symptoms have been linked to a worse decline in quality of life (Bega et al. 2015).

The main strengths of this study are its robust data collection methods and comprehensive analysis; it offers a well‐rounded understanding of the cognitive and neurobehavioral symptoms of PD by means of both objective and subjective measures. This research also contributes to the growing body of literature on the importance of taking sex into account while doing medical research, providing a novel insight into its association with motor characteristics in the clinical presentation of PD. Indeed, this is the first study to specifically investigate the interaction between sex, motor phenotype, and motor lateralization on PD non‐motor symptoms. An additional strength is the use of a heterogeneous sample population, including patients of different ages and genders, different disease durations, different onset phenotypes, and different motor lateralization, guaranteeing the generalizability of the findings across different PD demographics. However, the cross‐sectional design of the current study does not allow for evaluating changes over time. Another possible limitation might be the imbalance in the frequency of motor phenotypes in males and females, with 58% of females in the TD onset group compared to 45% of males, and the opposite trend for RA (42% in females, 55% in males). However, this is in line with previous literature showing a higher frequency of the TD phenotype in females than males (Haaxma et al. 2007; Solla et al. 2012).

Finally, the lack of a healthy group prevents determining whether the differences found are specific to PD or reflect cognitive and emotional patterns present in the population. However, we have control data for the Italian population on all tests, and we adjusted the patients’ scores based on the Italian normative data, overcoming this limitation. Future research should explore the longitudinal impact of sex on cognitive and neurobehavioral symptoms in PD. Increased research on sex differences in PD is essential: clinical guidelines should be updated to reflect the importance of addressing sex‐specific symptoms in PD management, to move toward an increasingly individualized medicine built to meet all patients’ needs.

## Author Contributions

**Massimo Favaro**: methodology, formal analysis, conceptualization, writing–original draft, investigation. **Chiara Longo**: writing–review and editing, investigation. **Donatella Ottaviani**: investigation, conceptualization. **Alessandra Dodich**: methodology, supervision, formal analysis. **Costanza Papagno**: Conceptualization, data curation, project administration, resources, supervision, writing–review and editing.

## Conflicts of Interest

The authors declare no conflicts of interest.

## Peer Review

The peer review history for this article is available at https://publons.com/publon/10.1002/brb3.70737

## Data Availability

The data supporting the findings of this study are available from the corresponding author upon reasonable request.
